# Traditional opal mining practice in Ethiopia, challenges and its economic impact on rural households: the case of wollo opal mining

**DOI:** 10.12688/f1000research.156436.3

**Published:** 2025-08-01

**Authors:** Tadesse Wudu Abate, Addise Zemelak Sisay

**Affiliations:** 1 Economics, Woldia University, Weldiya, Amhara, 400, Ethiopia; 2 Geology, Woldia University, Weldiya, Amhara, 400, Ethiopia

**Keywords:** Challenges, Economic impact, Endogenous Switching Regression Model, Wollo Opal, Ethiopia

## Abstract

**Background:**

The discovery spurred further exploration, leading to the expansion of opal mining into neighboring districts. Numerous cooperative groups, composed of small-scale miners, traditionally explore, develop, and extract significant amounts of rough opal gemstones. This study tries to investigate the challenges and economic impacts of traditional opal mining practice in wollo province of Ethiopia.

**Methods:**

The study used in this study is primary data collected from rural households, which are living in five districts of north wollo zone, in Amhara regional state. The data was collected using field observation, focus grouped discussion, interview, and questionnaire from sampled households. The study uses both descriptive and econometric methods of data analysis to achieve its objective.

**Results:**

Among the challenges of traditional opal mining, primitive way of exploring and extraction, limited access to market and low institutional support in terms of training and finance are identified. The probit model reveals that, increased education, access to training, and access to credit positively influence the likelihood of engaging in opal mining. The ESR model shows that, the average treatment effect on the treated (ATT) indicates that participating in Participation in opal mining is associated with higher monthly income by 31,380 ETB, while the average treatment effect on the untreated (ATU) shows a potential income increase of 31,625 ETB for non-miners if they engaged in mining.

**Conclusion:**

The study identifies three main categories of challenges faced by traditional opal miners: exploration and extraction issues, market limitations, and regulatory and institutional shortcomings. Exploration and extraction challenges include the lack of modern mining tools and knowledge, as well as difficult terrain, leading to inefficient and hazardous mining practices. Market-related challenges involve limited access to broader markets and lack of value addition, resulting in miners selling raw opals at significantly lower prices compared to polished ones.

## 1. Introduction

Ethiopia’s volcanic formation present significant opportunities for the extraction of metallic minerals, industrial minerals, and rocks and gemstone resources ( DEJENE, 2015). Amhara Mines agency has identified the occurrence of various metallic minerals, industrial minerals and rocks, gemstone and energy resources in different parts of the region associated with volcanic rock ( Ejigu, Ketemu, Endalew, & Assen, 2020). The Wollo plateau, part of northwestern Ethiopian Plateau, originated from the eruption of Ashange basalt, followed by the extrusion of Aiba basalts and Wogel Tena formation ( Wireko-Gyebi, Puwurayire, Nyamekye, & Akudugu, 2023). These volcanic episodes were characterized by extensional tectonic settings of continental flood basalt, which are marked by presence of bimodal volcanic suites ( Suryanarayana & Seid, 2022). Therefore, much of the mineral resources particularly peculiar gemstones are found associated with these volcanic rocks ( Chauviré et al., 2023).

The Arstesian Opal Mining (ASM) is a common global practice for extracting base and precious minerals ( Alves, Ferreira, & Araújo, 2021). Ethiopia is known by its traditional gold and gemstone mining from the biblical age and continues practicing up-to-date ( Alves et al., 2021). In the history of opal trade, Ethiopian opals were once exported to the ancient Kingdom of Israel ( Adesugba, 2018), which supports the notion that artisanal miners in Ethiopia have been extracting gemstone deposits for thousands of years. This indicates mainly the artisanal miners have mined the gemstone deposits in Ethiopia for several thousands of years. The mining sector contributes approximately 10% to the country’s foreign exchange earnings, with artisanal mining accounting for over 65% of this contribution ( Krishnan & Bhatia, 2009). Additionally, it provides employment to about 1.26 million people and supports the livelihoods of over 7.5 million individuals in the country ( Getaneh & Shikur, 2022; DEJENE, 2015).

**Figure 1.**
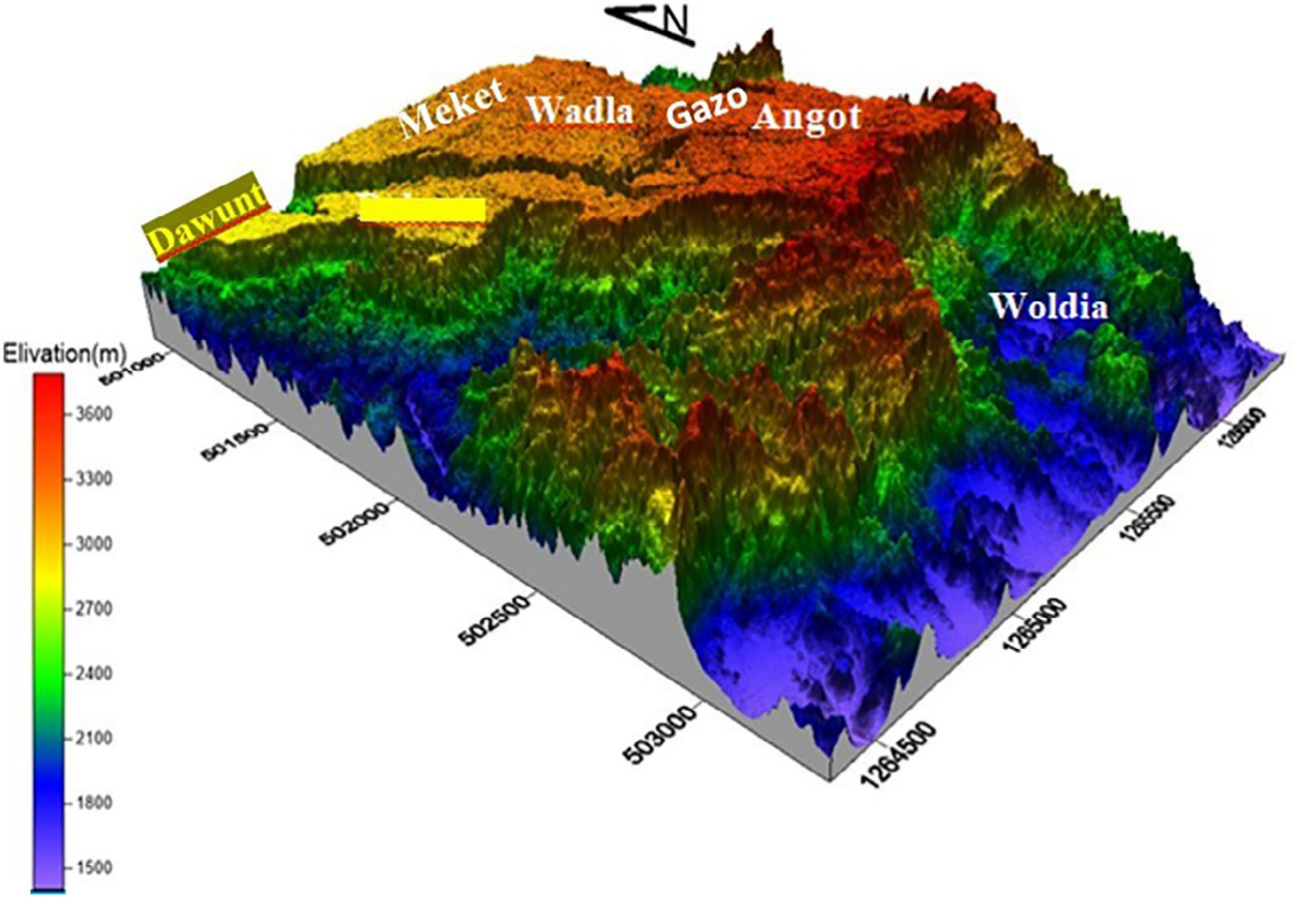
Map of the study area, the Wollo opal site.

Opal mining in Ethiopia is particularly renowned in the Wollo province, specifically in areas such as Wegeltena, Dawunt, Meket, Wadla, Gazo, and Angot (see Figure 1). Since the discovery of play-of-color opal in 2008 near Wegel Tena, Wollo has gained international recognition as a significant source of this gemstone ( Egunyu & Boakye-Danquah, 2024) The discovery spurred further exploration, leading to the expansion of opal mining into neighboring districts. Numerous cooperative groups, composed of small-scale miners, traditionally explore, develop, and extract significant amounts of rough opal gemstones. These miners control the distribution of rough opals, which are sold to gem dealers and eventually processed by cutters in Addis Ababa ( Herrmann & Maas, 2022). The production of opals in Wollo has been notable, with over 1,500 kg of rough opal extracted by 2010. As a result, opal mining has become a vital source of employment and economic activity in the region, attracting traders and researchers alike. However, several challenges, such as inadequate mining techniques and poor infrastructure, continue to hamper the potential of artisanal opal mining in Ethiopia. ( Katona & Krapf, 2020). Later on the discovery of opal resource expanded towards neighboring districts mainly Meket, Dawunt, Wadla, Gazo and Angot districts ( Alemu, 2018).

Ethiopia’s mineral resources, particularly opals, play a critical role ( Cartier, 2019) in its economic landscape. The discovery of high-quality opals in the mid-1990s and the subsequent breakthrough in 2008 positioned ( Rondeau et al., 2012) Ethiopia as a strong contender in the global opal market, challenging the dominance of Australia ( Mulaba-Bafubiandi, Ukponu, Odo, Singh, & Chauke, 2023). The opals from North Wello Delanta, known as Wello opals, are celebrated for their play-of-color and variety, leading to increased exports to major markets such as India, China, Europe, and the USA.

Ethiopia, renowned for its rich mineral resources, boasts a remarkable diversity of gemstones, with opals emerging as a key player in the trade sector ( Dawes, 2020). The discovery of opals in the mid-1990s initially highlighted Ethiopia’s potential in this market, but it was not until 2008 that a significant breakthrough occurred ( de Brito Barreto & Bretas Bittar, 2010). A substantial find of high-quality opal in North Wello Delanta, known as Wello opal, positioned Ethiopia as a serious competitor to the dominant Australian opal market. Wello opal, with its exceptional play-of-color and extensive color variations, quickly gained international acclaim, leading to a notable increase in exports to major markets including India, China, Europe, and the USA ( George, Whitten, Metters, & Abbey, 2022; Herrmann & Maas, 2022).

Small-scale mining is central to Ethiopia’s opal industry, particularly in North Wello Delanta, where artisanal methods are prevalent ( Onac & Forti, 2011). Despite the sector’s importance, challenges such as modernization and regulation persist ( Milkias, Demissie, & Meshesha, 2023). In 2013, the Ethiopian Ministry of Mines, Petroleum, and Natural Gas proposed legislation requiring ( Ofori, Takyi, Amponsah, & Gagakuma, 2023) that all opals be cut and polished domestically before export. However, this initiative faltered due to practical issues, including limited access to financing and a shortage of skilled lapidary workers ( Palgutová & Štrba, 2022; SUORINENI, 2022).

ASM practices in opal mining face numerous challenges, including the use of traditional mining techniques, low prices for unpolished opals due to the lack of local lapidary services, insufficient infrastructure, and inadequate financial and technical support. Despite these challenges, traditional opal mining has significantly improved the economic status of those involved.

Ethiopia’s mineral wealth is a cornerstone of its economic potential, with a notable array of gemstones contributing to its prominence in the global market. Among the diverse array of gemstones found within the country’s borders, opals have emerged as a significant asset but given little attention for its development. The journey of Ethiopian opals from their discovery to their status in the international market is both fascinating and complex, reflecting broader trends in artisanal and small-scale mining (ASM) and its impact on local economies ( Wireko-Gyebi et al., 2023).

This research aims to explore the challenges and economic contributions of small-scale opal mining in Wollo, Ethiopia. By examining the economic impacts on local communities, the study seeks to provide insights into the sector’s role in regional development. This includes analyzing factors such as employment generation, income levels, and local economic activities that are directly or/and indirectly related to opal mining. Additionally, the research will address the challenges faced by miners and offer recommendations for improving the sustainability and profitability of small-scale mining operations.

Understanding these dynamics is crucial for policymakers, stakeholders, and local communities to harness the full potential of opal mining while addressing the associated challenges. This study will contribute to the broader discourse on the role of small-scale mining in economic development and provide a case study that can inform similar contexts in Ethiopia and beyond.

## 2. Review of related literatures

### 2.1 Historical context of opal mining in Ethiopia

Opal mining in Ethiopia commenced in the mid-1990s with the discovery of opal deposits in Shewa Province ( Legasu & Chaubey, 2023). Initial assessments indicated that these opals did not match the quality of Australian opals ( Dawes, 2020), which had long dominated the global market. Despite these early challenges, a significant breakthrough occurred in 2008 with the discovery of high-quality opals in North Wello Delanta ( George et al., 2022). These opals, now referred to as Wello opals, exhibited exceptional characteristics, including a striking play-of-color and a broad spectrum of hues, quickly positioning them as strong competitors to Australian opals ( Habte et al., 2020). This discovery revitalized interest in Ethiopian opals and underscored the country’s potential to emerge as a notable player in the global gemstone market ( Kyngdon-McKay, Jorns, Wheat, Cushman, & Nemomissa, 2016).

### 2.2 The global market for Ethiopian opal

The international demand for Ethiopian opal has risen, with major buyers located in China and the USA ( Mustafa, Asfaw, Endris, & Bojago, 2023). However, this growing demand is accompanied by several challenges ( Senior & Deveson, 2023). A primary issue is the spread of misinformation ( Trickey, Lucifora, Wan, & Scott, 2013) regarding the qualities of Wello opals. Misconceptions associating Wello opals with the lower-quality Shewa opals have negatively affected their market value. This misinformation, combined with limited awareness and ineffective marketing ( Senior & Deveson, 2023), has resulted in Wello opals being priced lower internationally than their quality would warrant ( Ofori et al., 2023).

The Ethiopian government’s 40:60 rule mandates that a significant portion of opals be cut and polished domestically before export, aiming to promote local processing and add value to the gemstone sector ( Katona & Krapf, 2020). However, this regulation has faced criticism. Many international buyers report that the quality of local cutting and polishing does not meet global standards, forcing them to purchase lower-quality stones at higher prices ( Chauviré et al., 2023). Consequently, the rule has inadvertently imposed a financial burden on buyers who could otherwise process the stones more efficiently in their own facilities ( Herrmann & Maas, 2022).

### 2.3 Challenges facing the Ethiopian opal sector

The growth and profitability of Ethiopia’s opal mining sector face several hurdles. The local lapidary industry struggles with skill deficiencies ( Chauviré et al., 2023). Although some Indian traders have established cutting facilities in Ethiopia, the quality of local craftsmanship is often considered inadequate ( Katona & Krapf, 2020). This reliance on external expertise from Jaipur has exacerbated costs and constrained the sector’s competitive advantage ( Getaneh & Shikur, 2022). The 40:60 export rule, while well-intentioned, has not effectively fostered a robust domestic lapidary industry. The Ethiopian government’s emphasis on the size of rough and cut stones rather than their intrinsic qualities, such as play-of-color and clarity, has led to inflated valuations ( Qin, Yi, & Zhang, 2024). Additionally, the complexities of the export process, including the requirement for foreign currency deposits in Ethiopian accounts, create further barriers for exporters ( Legasu & Chaubey, 2023).

The domestic market for Ethiopian gemstones remains underdeveloped. The lack of local demand for opal jewelry, coupled with insufficient consumer and retailer knowledge about gemstones, poses a significant issue. The underdeveloped state of the domestic jewelry industry limits opportunities for local value addition. Illegal activities, including gemstone smuggling and unregulated lapidary operations, undermine the legitimate market ( Egunyu & Boakye-Danquah, 2024). The presence of a black market for lower-quality gemstones affects the sector’s economic growth and poses challenges for law enforcement and regulatory agencies ( Alves et al., 2021).

## 3. Method

### 3.1 Description of the study area

The target area is located in northern Ethiopia, Amhara regional state specifically ~550 km north of Addis Ababa, the capital city of Ethiopia (see Figure 1). Geographically UTM coordinates of 1284000N bound it to 1330000N and 501000E to 506000E latitude and longitude respectively. The area is accessible through Woldiya-Bahr Dar asphalted roads, and a number of gravel roads and footpaths generated by the local people and the administration. The north wollo zone has 13 rural districts and 5 urban city with diversified culture religion and climate. The zone is broadly classified in to lowland and highland districts based on alltitude. The target area of this study founds on the highland districts namely Wadlam Angot, Meket, Dawunt and Gazo ( Habte et al., 2020).

### 3.2 Methods of data analysis

Both exploratory and inferential research designs are used in this study. Since the challenges of Opal mining in north Wollo provinces are not studied yet, exploratory research design is applied to investigate the challenges that the traditional opal miners are facing. Inferential research design is applied to measure the economic impact of traditional opal mining on rural households. With this regard, mixed research design using both qualitative and quantitative methods of data analysis are applied. Descriptive and econometric methods of data analysis are also applied to investigate the challenges of economic impacts of traditional opal mining practices in wollo provinces ( Dawes, 2020).

### 3.3 Data type and methods of data collection

The type of data used in this study is primary data obtained from traditional opal miners of Wollo provinces in Ethiopia in 2024. The methods used to collect those primary data are, field observation also known as the desk study, questionnaire and interview. To gather data relating with the challenges of traditional opal mining, field observation, desk observation, focus group discussion and interview are applied. Finally to address the economic impact of practicing traditional opal mining, semi structured questionnaire is distributed to the sampled households and relevant information is collected ( Herrmann & Maas, 2022).

### 3.4 Sampling procedure

The study covers mining areas of North Wollo zone including five districts of Gazo, Wadila, Flakit, Dwunit, and Angot. Multi stage and stratified sampling technique are applied to get the sampling units, which are rural households. First, out of the 13 districts found in the North-Wollo administrative zone, five districts namely Gazo, Wadila, Flakit, Dwunit, and Angot are selected purposively based on high access to opal minerals. Then households of those five districts are classified in to two strata of participants and non-participants of traditional opal mining practices. Finally, each respondents are selected using systematic random sampling technique. The sampling techniques applied so far is important to collect data that helps researchers to analyze the economic impact of traditional opal mining practice. In addition to this, to investigate the challenges of opal mining in the study are only those respondents who are engaging in opal mining are selected as follows. In the study area there are about fifth eight legal association engaged in opal mining practices and two individuals, the leader and the secretory of each association are selected and interview and discussion are held on the challenges they are facing at the time of mining and post mining challenges relating to market linkages.

### 3.5 Sample size determination

Determining the sample size for a heterogeneous population often involves considering the variability within the population. For this purpose, we used Cochran’s formula, which is designed to provide a sample size that accounts for variability in a large population.

$$n_{0}=\frac{z^{2}p\left(1-p\right)}{e^{2}}$$

Where:

• n _0_ is the sample size.
• Z is the Z-score (the number of standard deviations a data point is from the mean) corresponding to the desired confidence level (e.g., 1.96 for 95% confidence).
• p is the estimated proportion of the population that has the attribute in question (if unknown, 0.5 is used as it provides the maximum variability).
• e is the desired level of precision (the margin of error).
$$n_{0}=\frac{1.96^{2}*0.5\left(1-0.5\right)}{0.05^{2}}$$

$$n_{0}=\frac{0.9604}{0.0025}$$

$$n_{0}=384$$

The sample for finite population can be obtained from finite population correction factor formula as follows;

$$n=\frac{n_{0}}{1+\frac{n_{0}-1}{N}}$$

Where:

• n is the adjusted sample size.
• n _0_ is the initial sample size from Cochran’s formula given to be 384 for 5% margin error.
• N is the population size.
$$\begin{array}{c} n=\frac{384}{1+\frac{384-1}{91078}}=382 \end{array}$$

This total sample is proportionally distributed to each districts based on their household size (as presented in Table 1).

**Table 1.** The proportional distribution of sample across districts.

Name of the district	Number of populations in the district	Number of rural households	Number of sample
Angot	61441	12288	52
Meket	161353	32270	136
Wadla	84802	16960	69
Dawunt	86109	17222	72
Gazo	61688	12338	53
Total	1137602	91078	382

Note: The total sample from each district is classified in to the two strata of opal mining participants and non-participant groups. Out of the total sample of 382 households, 21% respondents are taken from traditional opal miners and the remaining sample cover households that are not engaging in opal mining practices.

### 3.6 Model specification

In this study, we used two econometric models namely, the probit model and the Endogenous Switching Regression models. The first model, probit model uses to identify the determinants of households to participate in traditional opal mining and the second model, Endogenous Switching Regression model uses to investigate the impact of engaging in traditional opal mining on household income. Therefore, Logit and probit models are more appropriate for this type of binary dependent variable regression analysis. The different between the two is only the Cumulative distribution function they follows. By assuming the data used in this study to have a normal distribution, we prefer probit regression model which is specified as follows.

**3.6.1 The probit model specification**

Household status of being traditional opal miner is binary by its nature. Every household incorporates into two mutually exclusive groups of either being a member of traditional opal miner or being non-traditional opal miner.

$$\mathrm{yi}=\beta_{0}+\beta_{1}X_{1}+\beta_{2}X_{2}+\beta_{3}X_{3}+\beta_{4}X_{4}+---+\beta_{n}X_{n}+\epsilon_{i}$$ 1A

Where,

✓ *yi* is the opal mining participation status of household and given 1 if yes and zero if no
✓ *Xi* are explanatory variables that can affect the household’s participation status
✓
βi , represents the change in probablity of being opal miner when *Xi* changes
✓
$\epsilon_{i}$ is the error or disturbance term of the regression model

**3.6.2 The endogenous switching regression model**

The household participation in a traditional opal mining scheme is not random, as they self-select based on their perceptions of the benefits. Households choose to participant in opal mining only if they believe that they will received income in opal mining, which is higher than what they are actually obtaining agricultural practices.

The endogenous switching regression model tackles the issue by dividing individual household heads into different groups and examines the impact of these positions on outcome variable, which is annual income of households. The model consists of two components: the selection equation and the outcome equation. The selection equation focuses on the decision-making process or participation equation, while the outcome equation analyzes the relationship between the positions and the outcome variables.

The selection or participation equation is formed as:

$$I^{*}=\alpha Z_{i}+u_{i}\;\text{with}\;\left\{ \begin{array}{l} 1,\text{if}I^{*}>0\\ 0,\text{otherwise} \end{array}\right.$$ (1)

Where $I^{*}$ is the latent variable, which is not observable and expressed as a function of some observed household and institutional characteristics?

As already seen in the above equation, $I_{i}$ is a binary variable, which takes a value of ‘1’ for opal miners and ‘0’ for those who did not participate. $Z_{i}$ , represents factors that affect the participation decision and incorporates at least one explanatory variable which is not available in the regime equation. α , denotes the vector of parameters indicating the magnitude and direction of each explanatory variable’s effect on the decision to engage in opal mining practice. The residual $u_{i}$ captures the unobserved factors and measurement errors.

The outcome or regime equation on the other hand can be shown as;

$$r_{1}:\ln Y_{1i}=\left\{\mathrm{lnY}1i\right\}=\beta_{1}\;x_{1i}+\varepsilon_{1i}\;\text{if}\;I_{i}=1$$ (2)

$$r_{2}:\ln Y_{2i}=\left\{\mathrm{lnY}2i\right\}=\beta_{2}x_{2i}\pm \varepsilon_{2i}\;\text{if}\;I_{i}=0$$ (3)

Where, $\ln Y_{1i}$ and ${\mathrm{lnY}}_{2i}$ are the natural logs of income for opal miners and non opal miners respectively which are determined by the exogenous variables $\;X_{\mathrm{ji}}$ , $\;\beta_{1}$ and $\;\beta_{2}$ , are parameters that show the direction and strength of the relation between the outcome variable and the independent variables. In this research, annual income will be captured by household income (Y), $\ln Y_{1i}$ represents the natural logs of income for traditional opal miners and $\ln Y_{2i}\;$ represents the natural logs of income for non-opal miners groups.

The error terms of the above three consecutive equations ( equations 1, 2 and 3) are assumed to have a normal distribution with zero mean and covariance matrix,

where

$$\sum =\left[\begin{array}{ccc} \sigma_{u}^{2} & \cdot & \cdot \\ \sigma_{1u} & \sigma_{1}^{2} & {}^{*}\\ \sigma_{2u} & {}^{*} & \sigma_{2}^{2} \end{array}\right]$$

Where,

➢
$\sigma_{u}^{2}$ _,_ is the variance of the error terms in selection equation which is assumed to be one and coefficients are estimated up to a scale factor,
➢
$\sigma_{1}^{2}$ and $\sigma_{2}^{2}$ are the variance of the error terms in the outcome equation and
➢
$\sigma_{1u}$ and $\sigma_{2u}$ are the covariance of the participation equation and each outcome equation error terms.

On the other hand the * sign reflects the covariance of the error terms between each individual outcome equations since $\;Y_{1i}\;\text{and}\;Y_{2i}$ are not observed simultaneously.

The most important implication of the error terms structure is that, the correlation of error terms in the selection and outcome equations and their conditional expectation is non-zero

$$E\left[\varepsilon_{1i}/I=1\right]=\mathrm{Cov}\left(\varepsilon_{1i},u_{1i}\right).\frac{\varphi \left(\alpha Z_{i}\right)}{\Phi \left(\alpha Z_{i}\right)}=\sigma_{1u}.\lambda_{1i}$$ (4)

$$E\left[\varepsilon_{2i}/I=0\right]=\mathrm{Cov}\left(\varepsilon_{2i},u_{2i}\right).\frac{\varphi \left(\alpha Z_{i}\right)}{1-\Phi \left(\alpha Z_{i}\right)}=-\sigma_{2u}.\lambda_{2i}$$ (5)

Using this information the maximum likelihood estimation can be developed as;

$$\ln L_{i}=\sum_{i=1}^{n}I_{i}\left[\ln\varphi \left(\frac{\varepsilon_{1i}}{\sigma_{1}}\right)-\ln\sigma_{1}+\ln\Phi \left(\tau_{1i}\right)\right]+\left(1-I_{i}\right)\left[\ln\varphi \left(\frac{\varepsilon_{2i}}{\sigma_{2}}\right)-\ln\sigma_{2}+\ln\left(1-\Phi \left(\tau_{2i}\right)\right)\right]$$ (6)

According to Adesugba (2018), we can calculate the treatment effect on the treated, which is the main concern of policy makers, and it is the difference between the treated group and the counterfactual as well as the ATU as follows.

$$\mathrm{ATT}=E\left(Y_{1i}/I_{i}=1\right)-E\left(Y_{2i}/I_{i}=1\right)\Rightarrow X_{1i}\left(\beta_{1}-\beta_{2}\right)+\left(\sigma_{1u}-\sigma_{2u}\right)\lambda_{1i}$$ (7)

$$\mathrm{ATU}=E\left(Y_{2i}/I_{i}=0\right)-E\left(Y_{1i}/I_{i}=0\right)\Rightarrow X_{2i}\left(\beta_{2}-\beta_{1}\right)+\left(\sigma_{2u}-\sigma_{1u}\right)\lambda_{2i}$$ (8)

The base heterogeneity (BH _1_) for those households who are practicing traditional opal mining

$${\mathrm{BH}}_{1}=E\left(Y_{1i}/I_{i}=1\right)-E\left(Y_{1i}/I_{i}=0\right)\Rightarrow \left(X_{1i}-X_{2i}\right)\beta_{1i}+\sigma_{1u}\left(\lambda_{1i}-\lambda_{2i}\right)$$ (9)

For the group of households that who are not mining opal minerals, “the effect of base heterogeneity” is expressed as;

$${\mathrm{BH}}_{2}=E\left(Y_{2i}/I_{i}=1\right)-E\left(Y_{2i}/I_{i}=0\right)\Rightarrow \left(X_{1i}-X_{2i}\right)\beta_{2i}+\sigma_{2u}\left(\lambda_{1i}-\lambda_{2i}\right)$$ (10)

Finally, the effect called “transitional heterogeneity (TH)” estimates whether the effect of traditional opal mining is larger or smaller for households that are not included in the group of Opal mining. The difference between ( Equation 7) and ( Equation 8) gives us

TH=ATT−ATU (11)

This equation measures the difference between households who are engaged in traditional opal mining and those who are not practicing the traditional opal mining. Is also measures what will be the income of traditional opal miners if they were not engaged in opal mining practice and at the same time, it will calculate the income of non-opal miner households if they engaged in opal mining practices.

### 3.7 Description of variables and expected signs

The following table explained the variables, which determines the decision of households to engage to traditional opal mining. How those variables are measure, their nature of being continues or dummy and their expected signs to be positive or negative on the engagement of opal mining practice (see Table 2).

**Table 2.** Description of variables and their expected signs.

Variable name	Nature of variables	Description	Expected sign
Income	Continues	monthly income of households	The dependents variable of ESRM
Participation in opal mining	Dummy	1 if engaged in opal mining 0 otherwise	The dependent variable of probit model used to identify the determents of engagement in opal mining practice
Access to training	Dummy	1 if accessed 0 otherwise	+
Access to credit	Dummy	1 if accessed 0 otherwise	+
Education	Continues	Number of years the household head spent on education	+
Family size	Continues	The number of individuals with in a family	+
Marital status	Dummy	1 if married 0 otherwise	_
Land size	Continues	The size of the farm land owned by household’s as measured in hectare	_
Livestock	Continues	Number of livestock that the households owned	_
Awareness	Binary	1 if households have awareness on opal 0 otherwise	+
Market linkage	Binary	1 if households have market linkage 0 otherwise	+

Note: Awareness and market linkage are instrumental variable incorporated to capture the effect of unobserved factor in the Endogenous Switching Regression model.

## 4. Data analysis

Under this chapter, we analyzed data obtained from respondents using both descriptive and econometric methods. Additionally, we used qualitative and quantitate approaches to the objectives of the study. To address the objective of “investigating the challenges of traditional opal mining”, qualitative explanation of data obtained from field observation and focus group discussions are applied. Additionally, we applied econometric method of data analysis to capture the economic impact of traditional opal mining on rural households using Endogenous Regression model, in addition to the probit regression model, which investigates the determinants of engagement in traditional opal mining practice. The descriptive analysis is analyzed first using graphs tables and charts, and then Econometric model is addressed.

### 4.1 Challenges of traditional opal mining in North wollo

We analyzed the challenges of traditional opal mining by categorizing them into four key areas: issues with opal production techniques, constraints in value enhancement, obstacles in market dynamics, and difficulties within regulatory and institutional frameworks.

**Figure 2.**
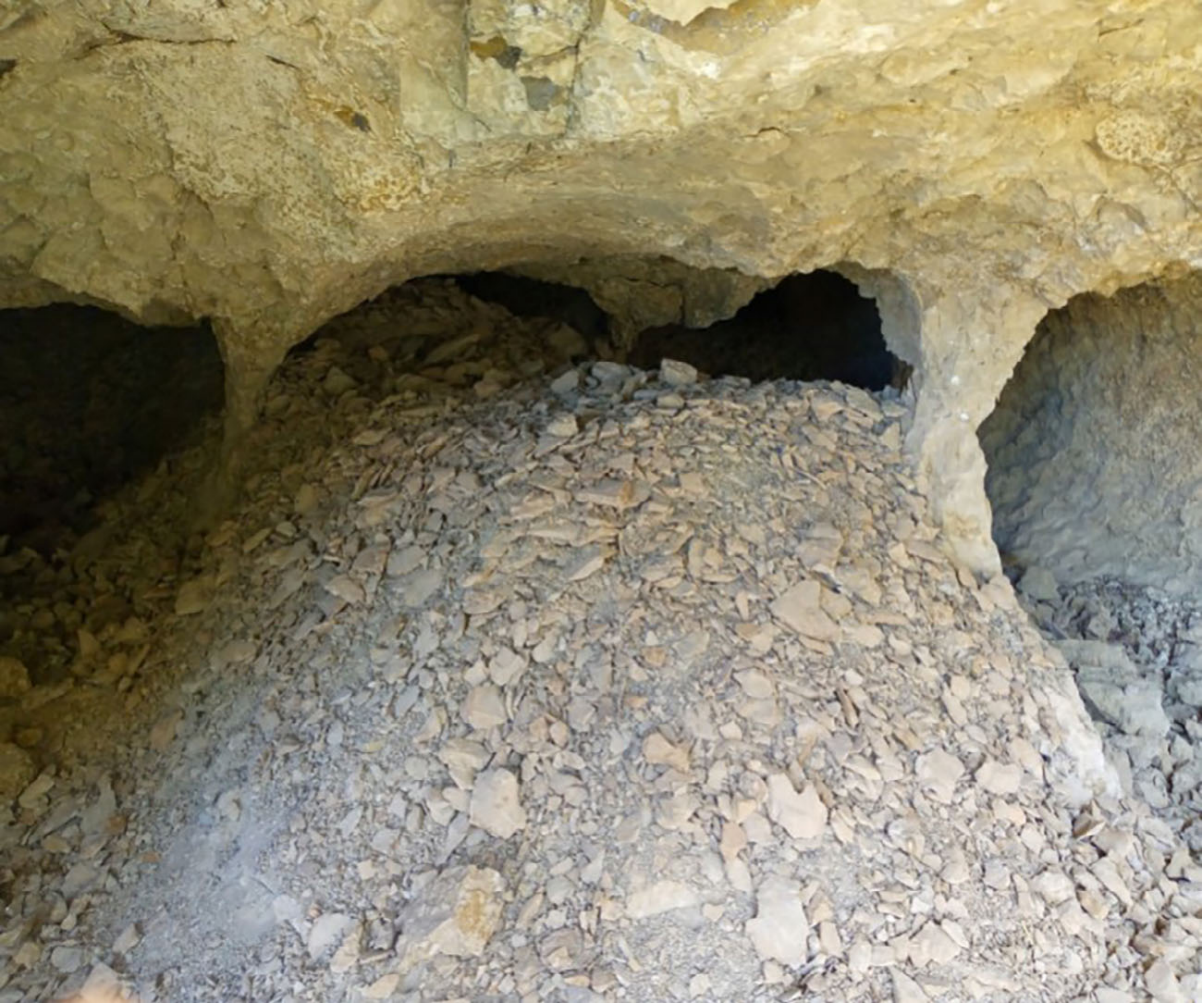
Opal Exploring and extracting process.

The first category comprises technical challenges encountered at the opal mining sites, including difficulties in exploration, extraction, and proper handling techniques. In the beginning, the opal gemstone in Wollo province was discovered by farmers near Wegel Tena, specifically in the Tsehay Mewcha area ( Rondeau, Mazzero, Bekele, Gauthier, & Fritsch, 2009). Subsequently, the discovery of opal resources expanded to neighboring districts, including Meket, Dawunt, Wadla, Gazo, and Angot districts. This led to the formation of numerous small mining groups that began exploring and extracting rough opal using traditional ways. Over the past seventeen years, opal exploration has continued using traditional methods, without proper training in exploration techniques or access to necessary gemstone searching equipment. The miners, all of whom are local residents, lack any geological background. They dig in various locations based on simple guesses, without understanding the geology of the area or the formations that host opal. This approach results in inefficient use of energy and significant time wastage. In addition to their traditional exploration activities, the miners rely on outdated opal extraction methods that expose them to significant risks. They dig trenches, pits, and adits to exploit opal, using homemade tools such as chisels, hammers, picks, and shovels ( Fakhouri et al., 2010). Most mining tunnels are situated on the steep slopes of ridges and mountainsides, requiring extensive digging into these challenging terrains ( Getaneh & Shikur, 2022). Unfortunately, this has led to numerous accidents, with many miners being buried in caves beneath sharp cliffs, resulting in loss of life. For instance, on February 8, 2024, over 20 miners were trapped in caves. Moreover, unsafe mining practices are predominant across most opal mining sites. Nearly all miners work without wearing essential safety equipment, such as helmets, goggles, masks, gloves, safety working shoes and cloth, or using proper ventilation. As a result, miners frequently suffer from injuries, severe headaches, and infectious diseases. Opal cracking is another significant issue that miners face during and after the extraction process ( Tsegaye & Ayalew, 2020). This occurs primarily due to the impact of traditional mining tools on the gemstone during burrowing. Furthermore, improper handling of immature opal after mining poses additional challenges for artisanal miners. These factors contribute to an imbalance between the miners’ efforts and the benefits they receive, often leading to financial strain and crises (see Figure 2).

**Figure 3.**
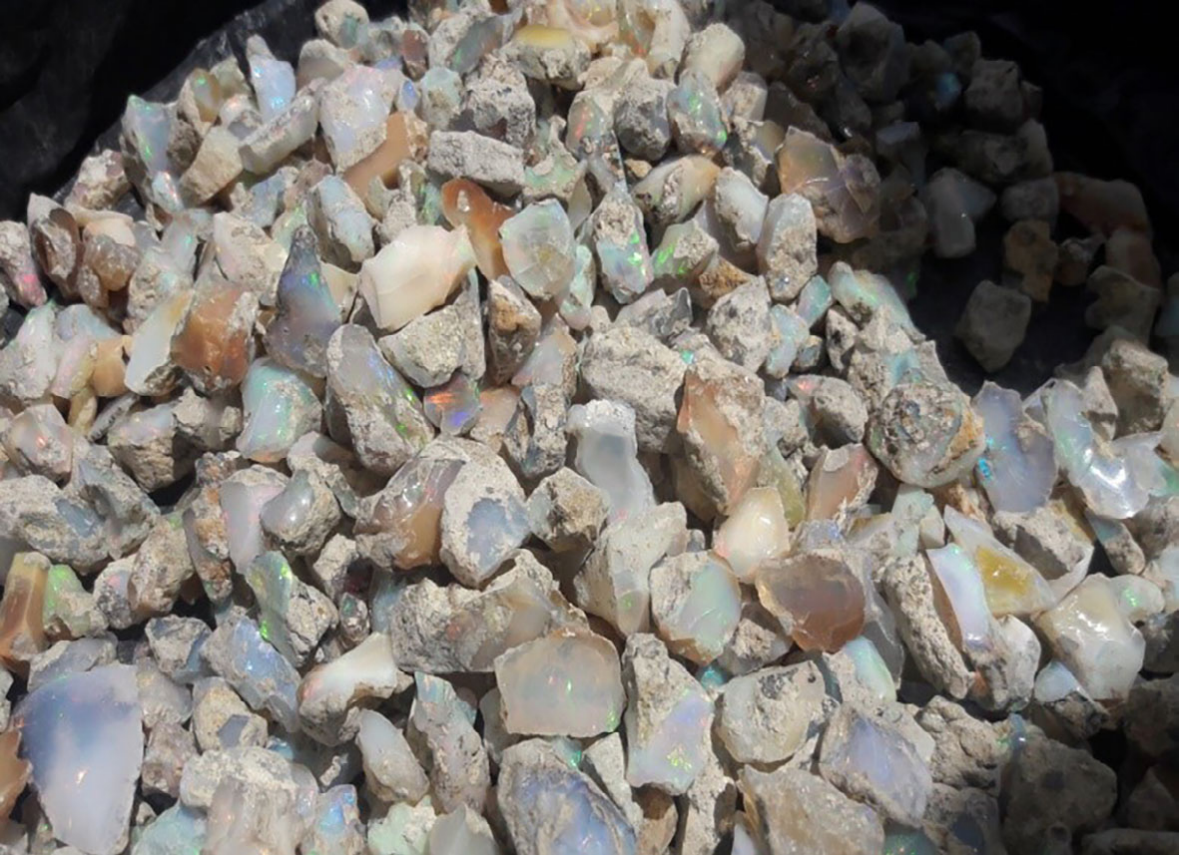
Wollo Opal before lapidary service.

The second-class addresses challenges related to value addition, such as the absence of awareness creation program, lapidary training centers, and lapidary machines that could enhance the appearance of raw opal, thereby boosting its market demand and economic value. In the past, miners sold their rough opal products without adding value, and this practice continues to the present day. Our study from respondents indicates that market value of rough opal is significantly lower compared to processed or polished opal. A kilogram of raw opal costs between $720 and $1,080, with an average of $900, while processed opal can rise between $3,240 and $4,680, averaging $3,960. This reflects an increase in value by $2,520 to $3,600 after processing, or about 4 to 4.5 times the original price. Despite this substantial potential for value addition, most opal mining associations lack lapidary machines and enhancement techniques, apart from a few individuals who have received training through the MEDA project. Consequently, artisan miners typically sell their raw opal to lapidaries and smugglers instead of processing it themselves (see Figure 3).

The third group highlights market-driven issues, including pricing complexities, market access, demand fluctuations, and distribution channels that affect the profitability and viability of the opal mining industry. A significant challenge for local miner associations is the lack of market linkage with legal exporters. Nearly all associations do not have market affiliation with legitimate opal traders and instead sell their products to brokers and local lapidaries at low prices. Consequently, miners receive inadequate compensation for their products, while smugglers benefit from these opal mining activities. Another challenge faced by miners, particularly those from the Meket area who are distant from Istayish and Delanta, is the lack of access to nearby markets. Additionally, these miners are often unaware of global opal prices and thus sell their products at relatively low prices, ranging from $250 to $667 per kilogram depending on quality. In contrast, the international market values these opals between $1,000 and $1,833 per kilogram. Furthermore, those associations have no awareness about the world’s opal price; they sell their product with relatively low costs ranged from 15,000 to 40,000 ETB per kilogram of precious opals depending on the opals quality. However, in international market it proper value is extended to 60,000 to 110000 ETB. Consequently, smugglers or local lapidaries, leaving miners at a disadvantage and effectively reducing them to laborers who do not benefit fairly from their efforts, typically determine the opal’s value. A previous study at the neighboring Delanta opal mining site revealed that smugglers (illegal sellers) and exporters were the primary and secondary beneficiaries in the opal market, capturing 36% and 28% of the profits, respectively ( Ayalew et al., 2020). In comparison, cutters, miners, and legal sellers accounted for only 8%, 12%, and 16% of the market profits, respectively. Similar patterns are observed at the Meket, Wadla, Angot, and Gazo opal mining sites, highlighting how the opal market system remains a critical issue in these areas. According to statistics from the focus group discussions, brokers purchase opals at very low prices without legal trade authorization. The opal miners of North wollo sold the extracted opal in local market for brokers at low cost since they have no any linkage to national and international markets. Instead of polishing and adding market value to their opal, they sold the raw opal without any quality improvement at low cost ^1^.

Lastly, we assessed challenges related to regulations and institutional frameworks, encompassing the legal, policy, and organizational barriers affecting the sustainability and growth of the opal-mining sector. According to the North Wollo zone mining development office, 32 licensed opal-mining associations in the districts of Meket, Wadla, Gazo, and Angot were officially registered. However, many groups and individuals remain unlicensed, fail to follow mining office guidelines, and do not pay the required taxes. Moreover, some illegal miners have resorted to violence, using weapons to clash with licensed miners and government officials. Although some legal miners pay taxes, many do not, leading to inconsistencies between licensed and unlicensed groups. Consequently, some licensed miners may resort to illegal practices due to the lack of support from government bodies. To address these issues, universities, federal and regional mining offices, and other NGOs should focus on creating awareness, providing training, and offering support to both legal and illegal miners. In 2024, Woldia University began offering training and material support, including mining and safety tools and a few lapidary machines, to some licensed associations. However, additional efforts and support are needed to achieve effectiveness of opal miners.

### 4.2 Descriptive analysis

The descriptive summary of both the categorical and continuous variables are analyzed using measures of mean, minimum, maximum, standard deviation, frequency, percentage, skewness and kurtosis measures as displayed in the two tables presented below.

The descriptive Summary of categorical variables (see Table 3) shows the proportion of the total sample engaged in traditional opal mining practice and their access to credit and training. Accordingly, out of the total sample of the study about 21.29% are engaging in traditional opal mining practices and sustaining their life using income obtained from the sales of opal gemstone. Out of those Artesian small-scale opal miners, 31.4% and 42.8% obtained support in the form of training and credit provision respectively. This shows that little attention is given to support small scale opal miners through proving training support relating to how to explore and extract the raw opal, how to add the value of opal applying lapidary, creating awareness about the international marker for opal mineral. That is why the opal miners in north wollo are selling one kilo gram raw opal for selling price of 40,000 to 50,000 ETB ^2^, while one kilo gram polished opal is sold for 250,000 to 300,000 ETB in Addis Ababa which the capital city of the country. Because of this, majority of the benefit of opal mining practice is taken by the illegal agents and broker involved in the opal and other mineral market.

**Table 3.** Description of binary variables of the study.

Variables	Type of variable with category	Numbers of obs.	Percentage	Chi-Squared test (p>chi ^2^)
Engagement in Traditional Opal Mining	Participants =	382	21.29%	0.002
	Non-participants = 1		78.71%	
Marital Status	single = 0	382	21.8%	0.418
	Married = 1		78.2%	
Access to Training	Accessed = 0	382	31.4%	0.000
	Otherwise = 1		68.6	
Access to Credit	Yes = 0	382	42.8%	0.000
	No = 1		57.2%	

The above table (see Table 4), shows the summary of continues variables of the study using measures of variation and central tendency such as mean, standard deviation, skewness, and kurtosis analysis. Land size and numbers of livestock have a respective mean value of 36.13 and 8.67 from which the distribution of land size is skewed to the right while number of livestock is skewed to the left. Education level of households’ ranges from 0 to 17 ^3^ indicating that the farmers’ education level shows high variance from illiterate farmers to fourteen years of education to mean that individuals who completed their secondary and preparatory schools are practicing traditional opal mining practices. The skewness and kurtosis values needs more explanation since those values are helpful to understand the distribution of observations to be either normal or not. A negative Skewness value indicates that variables are skewed to the left and positive values tells the present of positively skewed distribution.

**Table 4.** Description of continues variables of the study.

Variables	Type	Mean	Std.	Min	Max	Skewness	Kurtosis
Land size	Continues	36.13	10.6	20	61	0.69	2.43
Numbers of Livestock	Continues	8.67	14.38	0	16	-0.80	2.26
Education level	Continues	7.5	2.5	0	17	0.30	3.20
Family Size	Continues	4.2	1.9	1	8	0.57	1.78
Annual Income	Continues	5614	49.2	2970	18380	0.81	1.7

The kurtosis value of for education is more than three (leptokurtic distribution) to mean that few observations lied in the middle of distribution (around the mean) and more observation has an extreme education status of very high level and low status or being illiteracy level. The remaining variables income, family size, livestock and size of arable land has a kurtosis less than three (platikurtic distribution) tell us the distribution of less of those observations lies on the outliers than the normal distribution.

### 4.3 Econometric analysis

Under this part of the research, variables that can affect the household’s decision to engage in traditional opal mining practice are analyzed using probit regression model. The marginal effect results of all explanatory variables are given below (see Table 5) and the effect of each variable on the probability of being participant in traditional opal mining is analyzed.

**Table 5.** Marginal effect results of the probit model.

Variable	dy/dx	Std.Err.	Z	p >z
Household’s Land size *	-0.0235063	.00612	-3.84	0.000
Family size	0.0000889	.00155	0.06	0.954
Access to training *	0.2240494	.0586	3.82	0.000
Numbers of livestock *	-0.126319	.03161	-4.00	0.000
Access to credit *	0.325243	.05616	5.79	0.000
Marital status	0.0072583	.04659	0.16	0.876
Education level *	0.1944466	.04515	4.31	0.000

* Indicates that the variable is statistically significance at 1% level of significance.

As presented in the above table ( Table 5), the marginal effect result of the probit model shows that how the explanatory variables included in the model can affect) the probability of participating in traditional opal mining practices. There for, the effect of each explanatory variables is analyzed as follows after considering their statistical significance ^4^.

The size of arable land that the households owned has a negative impact on the probability of engaging in traditional opal mining practice. When the size of arable land owned by household farmers increased by one unit, the probability of being under the opal mining participant group decreases by 0.023. This shows that, rural households who have vast size of land are less likely involve in traditional opal mining since the agricultural income they are receiving from crop production is enough for them to sustain their life. ^5^ Similar with this, number of livestock’s owned by rural household farmers affect the engagement status of traditional opal mining negatively; increased number of households by one unit reduces the probability of being participants of opal mining by 0.126. This shows that individual household farmers that have higher number of livestock are less likely to participate in opal mining practices. This result is supported by many researches ( Chauviré et al., 2023; Getaneh & Shikur, 2022; Katona & Krapf, 2018; Legasu & Chaubey, 2023), which found inverse relationship between owning assets like land and livestock with participation in traditional mineral mining practices.

The remaining independent variables of the probit model, level of education access to training and access to credit affect the participation of opal mining positively. This result is supported by Slawson & Moffat (2020); SUORINENI (2022) and Wireko-Gyebi et al. (2023) More specifically, when level of education increases by one year, the probability of household famers to engage in traditional opal mining practice increased by 0.194. This indicates that majority of the traditional opal miners are educated individuals and hence educated individuals are more likely to be opal miners of the study area. Access to credit and access to training affect the membership of traditional opal mining group positively. The probability of individuals who accessed training have higher probability than that of their non-training accessed counter parts by 0.22. The probability of farmers who accessed credit is higher than household farmers who have not accessed it by o.33. Therefore, when households have access to credit that enabled them to purchase traditional tools used for mining and accessed training on how to extract the opal mineral using those tools easily are highly participating in the artesian small- scale opal mining activities in wollo provinces. This finding is supported by Rondeau et al. (2012); Milkias et al. (2023); Wireko-Gyebi et al. (2023) and Habte et al. (2020).

In addition to the probit model, which is used to identify the determinants of engagement status to traditional opal mining, the following table (see Table 6) presents the result of endogenous switching regression model, used to analyze the economic impact of traditional opal mining practice on rural householdsThe likelihood ratio test was significant at 5% level of significance, indicating that that the three equations, the opal mining participation equation and the outcome equations for two categories are jointly dependent. This implies that the model faced indigeneity problem and in this case; the ESR estimates are more accurate to study impact analysis than other model such as the PSM approach ( Mustafa et al., 2023).

**Table 6.** The estimation result of Endogenous Switching Regression Model.

Variables	Income for both category households			Income for traditional opal miners			Income for non-no traditional opal miners		
	Coef.	Std.Err.	P>z	Coef.	Std.Err.	P>z	Coef.	Std.Err.	P>z
Land size	-0.02350	.00612	0.000	-0.056	0.003	0.000	-0.05236	.00612	0.000
Marital status	0.00008	.00155	0.954	-0.012	0.038	0.751	0.06501	.02158	0.520
Education	0.2240	.0586	0.000	0.031	0.028	0.002	0.62400	.009068	0.000
Family size	-0.12631	.03161	0.000	-0.049	0.040	0.000	-0.08120	.002350	0.625
Access to training	0.32524	.05616	0.000	0.163	0.098	0.000	0.04320	.01216	0.000
Number of livestock	0.00725	-.04659	0.876	0.003	0.002	0.009	0.08702	-.026159	0.001
Access to credit	0.19444	.04515	0.000	0.029	0.069	0.018	0.41182	.05150	0.000
Awareness	-0.259	0.168	0.000						
Network to market	8.160	1.201	0.000						
_cons	1250	452.8	0.000	112.65	325.2	0.000	2120	237.16	0.000
sigma_1	0.532	0.021	0.000						
sigma_2	0.485	0.022	0.000						
rho_1	0.821	0.135	0.000						
rho_2	0.231	0.272	0.000						
LR test chi2 (1) = 7.98 Prob > chi ^2^ = 0.0031		Wald test of indepndent equatioss Prob > chi ^2^ = 0.0000			Wald test of model adequacy Prob > chi ^2^ = 0.0000				

Note: This table shows the Economic impact of traditional opal mining on rural households using results obtained from estimating the endogenous switching regression model.

In the selection (probit model) regression, different post estimation tests have been applied to check the validity of the model. With this regard, the result of the Wald test ( Watson & Teelucksingh, 2002) with p value equals with 0.0000 rejects the null hypothesis that states, “All regression coefficients are together equal to zero”. This shows that the model passes the overall significant test to indicate the presence of at least on significant variable to affect household’s engagement in opal mining.

VIF for continuous for explanatory variables have applied (see Table 8) to test multi-collinearity problem and the result given by mean vif values less than 10 shows that there is no serious multi-collinearity problem among the explanatory variables. In addition to this, we applied robust regression to control the effect of heteroscedasticity problem on estimation result. The covariance between the participation equation for engagement in opal mining and the outcome equations for household income was found to be non-zero, indicating the presence of endogenous switching in the model as stated in ( Bidzakin, Fialor, Awunyo-Vitor, & Yahaya, 2019) and this makes the rationality of using the ESR model.

The correlation coefficients between opal mining participation equation and the income of engagement and non-engagement are both negative ( Tesfay, 2020). This negative correlation coefficients suggest that there are unobserved factors ( Angrist, Imbens, & Rubin, 1996) influencing both the decision to participate in traditional opal mining practice which implies that households with higher unobserved factor (awareness) are more likely to engaged in opal mining.

The below table (see Table 7) estimates the effect of traditional opal mining on household’s income using an Endogenous Switching Regression model. The table presents the actual and counterfactual income and the average treatment effect ( Dawes, 2020) on treated (ATT) and untreated (ATU), which then helped to calculate the transitional heterogeneity, which measures the difference income between the traditional opal miners in the study area ( Katona & Krapf, 2018).

**Table 7.** The impact of traditional opal mining participation on household income.

Alternatives	Decision stage		Effect of being opal miner on income
	Participate in opal mining	Not-participating opal mining	
Traditional opal miners	36,250	4870	TT = 8264
Non- participants of opal mining	32,860	4625	TU = 4832
Heterogeneity effect			TH = 3432

**Table 8.** VIF result of variables.

Variables	Vif	1/vif
Training	1.64	0.608
Access to credit	1.62	0.606
Land size	1.30	0.769
Family size	1.18	0.847
Education	1.05	0.949
Livestock	1.02	0.976
Marital status	1.02	0.980
Income	1.00	0.996
Mean vif	1.23	

More specifically, the average monthly income for traditional opal miners is estimated to be 36250 ETB, but would be decreased to 4870 if they were not participating in the opal mining practice. On the other way, households who are not participating in the traditional opal mining are earning monthly income of 4625 ETB, which can be increased to 32860 ETB if they are treated and engaged in the traditional opal mining practices agrees with the findings of George et al. (2022). Those differences are the effect traditional opal mining on the treated and non-treated groups. If the treated groups were untreated, 31380 will lower their income while the income of untreated will increased by 31625 if they were treated and engaged in the traditional opal mining practices similar with the results of Herrmann and Maas (2022). In addition to this, the results of transition heterogeneity ^6^ confirm that the engagement in traditional opal mining leads to higher monthly income when compared with those who are not participating in opal mining in wollo provinces. The monthly income of households who engaged in traditional opal mining practice had higher income than that of non-opal miner households by 3432 ETB. This means household farmer who engaged in opal mining would have lower benefits if they do not participate in mining, while farmers who do not engaged in opal mining will have higher benefits if they do engaged in opal mining practice. This result is consistent studies conducted by Mustoe (2023); Habte et al. (2020); Ejigu et al. (2020); DEJENE (2015) and Alemu (2018).

In our ESR model, we used awareness and market linkage as instruments to identify the selection equation (participation in opal mining). The validity of these instruments is evaluated through two main criteria of relevance; both variables are statistically significant predictors in the selection equation, as shown by the first-stage regression. Specifically, market linkage has a strong positive correlation (r = 0.61, p < 0.01) and awareness has a moderately positive association (r = 0.39, p < 0.05) with the decision to engage in traditional opal mining. Exogeneity (Exclusion Restriction): These instruments are not included in the outcome equation (income) and are theoretically justified. Awareness of opal mining opportunities and market linkage increase the probability of engaging in mining but do not directly affect income unless the household actually participates in mining. Households not involved in opal mining are unlikely to benefit from market linkages related to opal, and awareness alone does not translate to income without participation. To further verify this, we conducted an overidentification test using the Sargan-Hansen test. The test statistic was χ ^2^(1) = 1.47, p = 0.22, indicating that the instruments are valid and uncorrelated with the error term in the outcome equation.

## 5. Conclusion and recommendations

### 5.1 Conclusion

This study is conducted to investigate the challenges and economic impact of traditional opal mining in north wollo zone of Ethiopia. The study identifies three main categories of challenges faced by traditional opal miners: exploration and extraction issues, market limitations, and regulatory and institutional shortcomings. Exploration and extraction challenges include the lack of modern mining tools and knowledge, as well as difficult terrain, leading to inefficient and hazardous mining practices. Market-related challenges involve limited access to broader markets and lack of value addition, resulting in miners selling raw opals at significantly lower prices compared to polished ones. Regulatory and institutional problems exacerbate these issues, with weak enforcement and inadequate support for miners.

Descriptive statistics reveal that a significant portion of the sample (21.29%) engages in traditional opal mining. However, access to training and credit is limited, contributing to low value addition and exploitation by intermediaries. The probit regression model indicates that larger land sizes and higher numbers of livestock negatively affect the likelihood of engaging in opal mining, while higher education levels, access to training, and access to credit positively influence participation.

The Endogenous Switching Regression (ESR) model shows that traditional opal mining significantly affects household income. On average, opal miners earn 36,250 ETB monthly, compared to 4,870 ETB if they did not mine. Conversely, non-miners could potentially earn 32,860 ETB if they engaged in opal mining. The transition heterogeneity analysis confirms that participation in opal mining leads to a higher income compared to non-participation, with a monthly income differential of 3,432 ETB favoring opal miners. The findings highlight the need for targeted interventions to address the challenges faced by traditional opal miners. These include improving access to modern mining tools, enhancing market linkages, and strengthening regulatory frameworks. Additionally, providing training and credit support can improve the efficiency and profitability of opal mining. Addressing these issues will not only enhance the economic benefits for miners but also contribute to sustainable development in the region. Overall, while traditional opal mining presents significant opportunities for income generation, addressing the identified challenges is crucial for maximizing its economic benefits and ensuring the well-being of mining communities in North Wollo.

### 5.2 Recommendations

Based on the findings of the study, we recommend the following ideas to address these challenges and unlock the full potential of Ethiopia’s opal mining sector.

➢ **Training and Capacity Building:** Enhancing the skills of gemstone miners and lapidaries is critical. Training programs should focus on modern mining techniques, operational health and safety, and gemstone valuation. Additionally, business training for mining cooperatives can help improve productivity and resilience.
➢ **Financial Support:** Improving access to financing for small lapidaries and exporters is essential for enhancing competitiveness. A robust marketing strategy should be developed to communicate the unique attributes of Ethiopian opal to both domestic and international consumers. Long-term strategies could include promoting the sale of cut and polished opals directly to global markets and developing niche specializations within the sector.
➢ **Enhancing Cutting and Polishing Skills:** Establishing partnerships between lapidary training centers and the gemstone industry can improve domestic cutting and polishing standards. Funding should be increased for training institutes to ensure sufficient practice materials and to support the development of high-quality cutters.
➢ **Addressing Illegal Activities:** Investigating illegal gemstone supply chains and enhancing enforcement measures are crucial for protecting the legitimate market. Developing a comprehensive framework for addressing illegal activities and improving customs control can help mitigate the impact of smuggling.
➢ **Infrastructure and Policy Improvements:** Improving infrastructure, such as roads and gemological institutes, can enhance the efficiency of gemstone trading and processing. Revising policies related to export prices and taxes could also support the growth of the domestic cutting and polishing sector.

#### List of declarations

##### Ethics and consent

This study, titled “Traditional Opal Mining Practice in Ethiopia, Challenges, and Its Economic Impact on Rural Households: The Case of Wollo Opal Mining,” was conducted in accordance with the ethical principles outlined in the Declaration of Helsinki. The research received ethical approval from the Research and Community Service Bureau of the College of Business and Economics at Woldia University on May 10, 2024, under approval number RCSTT/184/2024. The following ethical standards were adhered to throughout the research process:

1. **Compliance with the Declaration of Helsinki:** The study was conducted in line with the principles of the Declaration of Helsinki, which emphasizes the protection of human participants, respect for their rights and dignity, and the promotion of well-being. This research prioritized participants’ health, welfare, and confidentiality.
2. **Informed Consent:** All participants were fully informed about the objectives, methods, risks, and potential benefits of the study. Participation was voluntary, and verbal informed consent was obtained from each participant prior to their involvement. Participants were also made aware of their right to withdraw from the study at any time without consequences.
3. **Confidentiality and Data Protection:** Personal data was handled in strict confidence. Participant identities were anonymized, and no personally identifiable information was disclosed in the final analysis or publications. All data were securely stored, and only the research team had access to them. Data protection protocols were followed to prevent unauthorized access.
4. **Non-Coercion and Autonomy:** No participant was coerced or pressured into participating. Autonomy was respected throughout the study, and participants were encouraged to make informed decisions regarding their participation. The research ensured that vulnerable groups, such as women or economically disadvantaged individuals, were not subjected to any form of exploitation or undue influence.
5. **Protection of Vulnerable Populations:** The study took particular care to protect vulnerable individuals. No minors were involved in the study. “In this study, participants provided verbal consent after being fully informed about the research’s purpose, procedures, and potential risks. Verbal consent was documented as per ethical guidelines, ensuring participants understood their voluntary involvement and right to withdraw at any time. Institutional approval was obtained to use verbal consent in place of written forms.” The reason for applying this verbal consent was the presence of some illiterate respondents (local farmrers) which are involving in the this study.
6. **Minimizing Harm:** The research team made every effort to minimize any potential risks or harms, both physical and psychological, to participants. Interview questions were designed to be non-intrusive, and participants were assured that they could skip any questions that made them uncomfortable. Should any participant experience distress, protocols were in place to provide support and discontinue the interview if necessary.
7. **Transparency and Feedback:** Participants were informed about how the research results would be used and how they could access the findings if desired. The results will be disseminated to local communities, policymakers, and other relevant stakeholders, ensuring that the research findings contribute to improving the livelihood and economic well-being of rural households involved in traditional opal mining.

## Authors contributions

1. **TWA:** Generate the idea about the research title, problem statement, research objectives; write a literature review, data collection, data analysis, and recommendation.
2. **AZS:** Literature writing, data analysis and recommendation.

## Data Availability

the data used in this study is underline data fully available on respiratory called Zenodo and the DOI number for the data is stated here and on the online submission with the title traditional opal mining practice in Ethiopia, challenges and its economic impact on rural households: case of wollo opal mining obtaining license from Creative Commons Attribution 4.0 International. This data can be cited as “Abate, T. W. (2024). Traditional opal mining practice in ethiopia, challenges and its economic impact on rural households: case of wollo opal mining. Zenodo. Or can be accessed with DOI: https://zenodo.org/doi/10.5281/zenodo.13739221. This project contains: Interview data for opal.xls Data are available under the terms of the Creative Commons Attribution 4.0 International license (CC-BY 4.0). Extended data for this study is the interview guide line uploaded on Zenodo with title “Interview guide line for Wollo opal mining” with Doi number of https://doi.org/10.5281/zenodo.13856212. This extended data obtaining license from Creative Commons Attribution 4.0 International. It can be cited as “Tadesse Wudu, A., & Addis Zemelak, S. (2024). Interview guide line for Wollo opal mining. Zenodo. https://doi.org/10.5281/zenodo.13856212”. This project contains: inretview guide line.xlsx Data are available under the terms of the Creative Commons Attribution 4.0 International license (CC-BY 4.0).

## Footnotes

^1^
In June 2025 one USD is exchanged for 133 ETB and hence the value of birr can be easily changed to dollar to make the unit of measurement similar.

^2^
ETB is to indicate the national currency of the country called “Ethiopian Birr”.

^3^
Among the traditional opal miners of the study area, few respondents are BSc degree holders who had been graduated in field of Engineering. Therefore, since 5 years of higher education is required to be graduate of engineering, some respondents have a maximum year of education equal to 17 years. However, majority of the respondents are either elementary school dropouts or illiterate farmers.

^4^
The statistical significant of variable is tested using z and p values adjacent to each variables. Using the rule of thumb, variables which have a z value higher than two of a probability value less than 5%(0.05) are statistically significant to affect the dependent variable. Following this rule, family size and marital status are statistically insignificant and hence no need of giving interpretation for the two variable. Other independent variables are tested to be statistically significant and their effect is interpreted based on their marginal effect results.

^5^
Household who are producing higher yield of crops fears the challenges of traditional opal mining, because they are sustaining their life easily without experiencing risk of life. As explained in the challenge of opal miners in wollo provinces previously, there and many challenges to engage in the mining practice, because of this, households who are relatively under good life sustaining economic activities does not prefer to engage in opal mining practice.

^6^

*The positive significant values for the transition heterogeneity effect (TH) of income indicates that the impact of traditional opal mining on income will be significantly higher for the participants relative to the non-participant households. In reverse to this, if the TH value is negative it indicates that then treated groups are earning lower income than the counterfactuals because of participating in opal mining practices.*

## Limitations and Directions for Future Research

This study focuses specifically on the traditional opal mining practices in five districts within the North Wollo Zone of Ethiopia. While the results provide valuable insights into the challenges and income-related impacts of mining in this context, the findings may not be directly generalizable to other opal-producing regions such as Delanta or Shewa, or to other artisanal mining sectors such as gold or emerald mining. Differences in geological conditions, infrastructure, institutional support, and market access across regions may lead to varying economic outcomes and policy needs. Therefore, caution should be exercised in extrapolating the findings beyond the study area. Future research should incorporate comparative studies across multiple mining zones, mineral types, and even across neighboring countries where artisanal and small-scale mining is prevalent. Such research would help better understand the heterogeneity of mining experiences and enhance the formulation of context-specific policy interventions.

## Software availability statement

This study used Stata version [stata 13] for the statistical analysis. Stata is a proprietary software available from StataCorp ( https://www.stata.com/). While Stata is widely used for econometric and statistical analysis, an open-source alternative that can perform similar functions is **R** ( https://www.r-project.org/) with packages such as **plm** (for panel data analysis), **MASS**, and **stats** (for econometric functions). R is freely available under the GNU General Public License.
