# Supplementary material for: Traditional opal mining practice in Ethiopia, challenges and its economic impact on rural households: the case of wollo opal mining
Source: F1000Res. 2025 Aug 1;13:1388. Originally published 2024 Nov 19. [Version 3] doi: 10.12688/f1000research.156436.3 (PMC12290443; doi:10.12688/f1000research.156436.3)
Supplement: Peer review history

# Reviewer report: Reviewer response for version 3

**DOI:** 10.5256/f1000research.185758.r402001

**Published:** 2025-08-28

**Authors:** Samy Andres Leyton Flor

**Affiliations:** 1 Charles Darwin University, Casuarina, Northern Territory, Australia

The authors have made visible efforts to address some of the core concerns raised in my earlier review, particularly in improving language clarity, expanding methodological explanations, and better linking results to the stated research objectives.

Is the work clearly and accurately presented and does it cite the current literature?

Partly

If applicable, is the statistical analysis and its interpretation appropriate?

Partly

Are all the source data underlying the results available to ensure full reproducibility?

Partly

Is the study design appropriate and is the work technically sound?

Partly

Are the conclusions drawn adequately supported by the results?

Partly

Are sufficient details of methods and analysis provided to allow replication by others?

Partly

Reviewer Expertise:

Ecological economics, environmental governance, and Indigenous knowledge systems to inform more inclusive environmental policy.

I confirm that I have read this submission and believe that I have an appropriate level of expertise to confirm that it is of an acceptable scientific standard.

# Reviewer report: Reviewer response for version 2

**DOI:** 10.5256/f1000research.183831.r393808

**Published:** 2025-07-24

**Authors:** Samy Andres Leyton Flor

**Affiliations:** 1 Charles Darwin University, Casuarina, Northern Territory, Australia

This is a **valuable contribution** to the literature on artisanal mining and rural livelihoods, particularly in Ethiopia. With substantive revisions to its methodology reporting, causal framing, and clarity, the manuscript can meet the standards of scientific rigour and policy relevance required for indexing.

 I encourage the authors to carefully address the following issues and resubmit for further consideration.

 A. **Instrumental Variable Validity (Must Address)**

- The ESR model relies on “awareness” and “market linkage” as instruments. The manuscript does not test or justify its **exogeneity** or **relevance**.
- **Recommendation**: Include statistical validation of instruments (e.g., correlation with outcome, overidentification test) and explain why they satisfy exclusion restrictions.

B. **Reproducibility and Method Transparency (Must Address)**

- No model syntax or estimation code is shared. Data structure is not explained in the manuscript.
- **Recommendation**:
  - Upload and cite analysis code (e.g., Stata .do files).
  - Provide a full data dictionary matching variables in the paper to the dataset columns.
  - Include the questionnaire and interview protocol (cited in the extended data) as appendices or summaries.

C. **Language, Clarity, and Consistency (Must Address)**

- Numerous grammar and syntax issues affect clarity. Some technical terms are misused (e.g., “lied in the middle” vs. “lay around the mean”).
- **Recommendation**: Conduct a professional language and technical copyedit. Focus on transition clarity, grammar correction, and removal of redundancy (e.g., duplicated history in the introduction).

D. **Overstatement of Causal Claims (Must Address)**

- The ESR model helps mitigate selection bias but does not fully establish causality. Claims like “opal mining increases income” should be reframed.
- **Recommendation**: Use more cautious phrasing (e.g., "associated with higher income after correcting for observable selection bias") and discuss residual endogeneity risks.

E. **Generalizability and Policy Relevance (Should Address)**

- The study draws broad policy conclusions from a localised sample. There is no comparison to other opal sites or mineral sectors in Ethiopia or beyond.
- **Recommendation**: Add a paragraph reflecting on the limitations of generalisation and suggesting areas for comparative research.

F. **Statistical Reporting and Diagnostics (Should Address)**

- Table 6 merges multiple models in a confusing format. Some standard errors appear mismatched. No confidence intervals are reported.
- **Recommendation**:
  - Separate the outcome equations into different tables.
  - Include 95% confidence intervals.
  - Add model diagnostics (e.g., pseudo R², AIC, normality of residuals).

Is the work clearly and accurately presented and does it cite the current literature?

Partly

If applicable, is the statistical analysis and its interpretation appropriate?

Partly

Are all the source data underlying the results available to ensure full reproducibility?

Partly

Is the study design appropriate and is the work technically sound?

Partly

Are the conclusions drawn adequately supported by the results?

Partly

Are sufficient details of methods and analysis provided to allow replication by others?

Partly

Reviewer Expertise:

Ecological economics, environmental governance, and Indigenous knowledge systems to inform more inclusive environmental policy.

I confirm that I have read this submission and believe that I have an appropriate level of expertise to confirm that it is of an acceptable scientific standard, however I have significant reservations, as outlined above.

# Response

**Published:** 2025-07-28

**Authors:** Tadesse Wudu Abate

**Affiliations:** Economics, Woldia University, Weldiya, Amhara, Ethiopia

Thank you for this important observation. In the revised manuscript, we have now included a justification for the choice of *awareness* and *market linkage* as instrumental variables based on both theoretical and empirical grounds. **Relevance:** We provide correlation evidence that these instruments are significantly associated with the selection equation (participation in opal mining) but not directly with the outcome equation (household income), satisfying the **instrument relevance** condition. **Exogeneity:** We argue that both *awareness* and *market linkage* influence household income only through participation in opal mining, thus meeting the **exclusion restriction** requirement. These variables do not directly determine income for non-participants. **Statistical Test:** We now report results from the overidentification test (e.g., Sargan test) and provide first-stage regression statistics to confirm instrument validity.

**B. **Thank you for pointing this out. In response, we have taken the following steps to improve reproducibility and methodological transparency: We have now uploaded the **Stata** **.do** **file** used for the full econometric analysis (probit and ESR models) to Zenodo under DOI: **[** 10.5281/zenodo.16525909 **]**, and cited it in the manuscript under *Software Availability*.

**C. **These important issues related to language clarity and terminology. We have thoroughly revised the manuscript to improve the overall readability and ensure technical accuracy. Specifically:

 A full **technical language edit** was conducted, focusing on grammar, syntax, and sentence structure to improve flow and comprehension. Incorrect technical terms and statistical expressions (e.g., “lied in the middle”) have been corrected (e.g., to “lay around the mean” or “clustered near the center of the distribution”). **Redundant historical context** in the introduction has been removed and merged into a coherent narrative to maintain focus and reduce repetition. Transitions between sections and paragraphs have been improved to guide the reader smoothly through the structure of the paper.

**D. **We acknowledge that while the Endogenous Switching Regression (ESR) model reduces selection bias due to observable characteristics, it does not fully eliminate potential bias from unobservable factors. In response, we have revised the manuscript to use more cautious and appropriate language. Statements such as “opal mining increases income” have been replaced with phrasing like “opal mining is associated with higher household income after controlling for observable selection bias.” We also added a limitation discussion acknowledging the possibility of residual endogeneity due to unobserved variables that may affect both mining participation and income.

 Relevant changes are made in the Abstract, Results, Discussion, and Conclusion sections to ensure consistency and methodological accuracy.

**E:**. We agree that the findings, while meaningful, are based on data from a specific geographic context the Wollo opal mining region in northern Ethiopia  and may not fully capture the diversity of mining practices or socioeconomic outcomes in other regions.

 In the revised manuscript, we have added a paragraph in the **Conclusion section** acknowledging the **limited generalizability** of our findings and the need for **comparative research** across different opal mining sites and other artisanal mineral sectors in Ethiopia and Sub-Saharan Africa. This addition strengthens the contextual framing of our policy implications and identifies important future research directions.

 F. thank you for your comment relating with the formatting of the table but since it was corrected based of the previous comments we preferred to be in this format.

# Reviewer report: Reviewer response for version 2

**DOI:** 10.5256/f1000research.183831.r393395

**Published:** 2025-06-25

**Authors:** Taşkın Deniz Yıldız

**Affiliations:** 1 Mining Engineering, Adana Alparslan Türkeş Science and Technology University, Adana, Turkey

**After the revision process, the authors improved the academic quality of their articles and** **they have made the necessary corrections** **. Congratulations to the authors. I recommend that the article be accepted for indexing**

Is the work clearly and accurately presented and does it cite the current literature?

Partly

If applicable, is the statistical analysis and its interpretation appropriate?

Yes

Are all the source data underlying the results available to ensure full reproducibility?

Yes

Is the study design appropriate and is the work technically sound?

Yes

Are the conclusions drawn adequately supported by the results?

Yes

Are sufficient details of methods and analysis provided to allow replication by others?

Yes

Reviewer Expertise:

AREA OF INTERESTS•          Mining legislation•          Mining exploration and operation projects•          Mineral economics, resources economics•          Costs and cost analysis, cost benefit analysis•          Natural resources rents, taxation•          Mining and energy/infrastructure investments conflicts•          Land acquisition and land use in mining, land use change, compensation, evaluation of abandoned mining areas in terms of environment and urbanism, industrial heritage•          Mining waste management, rehabilitation cost•          Occupational health and safety in mines, supervising of mines.

I confirm that I have read this submission and believe that I have an appropriate level of expertise to confirm that it is of an acceptable scientific standard.

# Reviewer report: Reviewer response for version 1

**DOI:** 10.5256/f1000research.171747.r352841

**Published:** 2025-01-23

**Authors:** Francis F Pavloudakis

**Affiliations:** 1 University of Western Macedonia, Kozani, Greece

The paper is very interesting as it deals with the effective exploitation of a mineral resource that is not adequately utilized to benefit mining enterprises, the local community, and the national economy of the country (Ethiopia).

 The structure of the paper is suitable for a scientific article, and the methodology applied has a solid scientific basis and is adequately described.

 However, the paper requires significant improvement in the presentation of all sections except the methodology section.

 Overall, the text needs more careful editing and improvement in the use of the English language. For example, in many instances, "Wollo district" is written without capitalizing the first letter. Is there a specific reason for this? Additionally, various errors such as "the study used in this study", "Arstesian Opal Mining", "in to", "fifth eight", "household size" instead of "number of households", "kilo gram", "there for", "number of households" instead of "number of livestock", "o.33", "vif instead of VIF", "income for non-no traditional opal miners", etc., need to be corrected.

 Furthermore, it would be preferable to integrate footnotes into the main text.

 Introduction and Literature Review: It seems that the authors have directly quoted information gathered from various sources without checking if some data are repeated. These two sections need to be rewritten.

 In addition, the opal production data are relatively old (2010). Are there more recent data available?

 Description of the Study Area: Although visually appealing, Figure 1 does not provide substantial information. Also, I understand that the horizontal and vertical scales are different. A simpler country map showing the position of the study area, the land morphology, and the major cities would be more informative.

 Model Specification: I do not understand why there is a reference to the Logit model since it is not used.

 Ethics Statement: This section should be omitted. Any relevant reference can be included in the accompanying text.

 Section 4.1: The content of this section does not seem to result from the methodology described earlier. Either the authors should describe how they arrived at the four key challenge areas and analyzed them, OR this entire section should be moved to the literature review OR, in my opinion, to a separate fifth section entitled: Discussion.

 Section 4.2: For comparison purposes, in the last paragraph, it would be useful to provide data on the average wages in the country and the region, as well as wages by sector of economic activity. Additionally, presenting the amounts in dollars would facilitate comparisons with other countries.

 In the same paragraph, why are references added? Do the amounts mentioned (Table 7) not arise from the analysis conducted in the current study?

Is the work clearly and accurately presented and does it cite the current literature?

No

If applicable, is the statistical analysis and its interpretation appropriate?

Yes

Are all the source data underlying the results available to ensure full reproducibility?

Yes

Is the study design appropriate and is the work technically sound?

Yes

Are the conclusions drawn adequately supported by the results?

Yes

Are sufficient details of methods and analysis provided to allow replication by others?

Yes

Reviewer Expertise:

Mining, Energy, Mine Land Reclamation

I confirm that I have read this submission and believe that I have an appropriate level of expertise to confirm that it is of an acceptable scientific standard, however I have significant reservations, as outlined above.

# Response

**Published:** 2025-01-27

**Authors:** Tadesse Wudu Abate

**Affiliations:** Economics, Woldia University, Weldiya, Amhara, Ethiopia

1. Dear Francis F Pavloudakis, Thank You For your Valuable comments that helped me improve the quality of my research. I have revised the articles by considering each of your comments. To make some issue clear,  we collected qualitative data using focus group discussion and this is used to analyzed the challenges of tradition opal mining practice in the study area and it is analyzed independent of the impact of traditional opal mining practice on households income, which is the second objective of the study and the data is collected from individual miners with more quantitative variables.
2. I have included ethics statement on the methodology part based on the request of the editors and I have removed it based on your comment.
3. For the map of the study, instead of using the study area, we preferred this geological map since it indicates the amount and distribution of  opal in the area. Thank You Sir.

# Reviewer report: Reviewer response for version 1

**DOI:** 10.5256/f1000research.171747.r343281

**Published:** 2025-01-03

**Authors:** Taşkın Deniz Yıldız

**Affiliations:** 1 Mining Engineering, Adana Alparslan Türkeş Science and Technology University, Adana, Turkey

This paper analyzes the problems of opal mining in Ethiopia and its impact on household incomes. There are very few studies on the effects of mining on household incomes. Therefore, this is a very valuable study that can serve as an example of practices in less developed countries. I think that if it is revised and published, it can fill an important gap in the literature. On the other hand, I think that the article should be improved in line with my suggestions below. In this direction, there are still deficiencies in the text of the article. I suggest that the article be given a "major revision" decision. I predict that the article will be highly cited if it is indexed after the revision.

**In “Introduction”**

 • *“(…) Wello opal, with its exceptional play-of-color and extensive color variations, quickly gained international acclaim, leading to a notable increase in exports to major markets including India, China, Europe, and the USA (George, Whitten, Metters, & Abbey, 2022; Herrmann & Maas, 2022).”*

 This paragraph has sentences in common with the paragraph above. Merge the two paragraphs and delete the repeated sentences.

 • *“Ethiopia’s vast mineral wealth, particularly its opals, is a cornerstone of its economic potential. The country’s journey from the discovery of opals to their prominence in the international market is both intriguing and complex, reflecting the broader trends and challenges in artisanal and small-scale mining (ASM).”*

 There are two similar sentences above. You can delete the ones above:

 • *“Therefore, the objectives of this research are*

- *Analyzing the challenges of traditional opal mining in Wollo provinces*

 

- *Investigating the economic impact of opal mining on the household in the study area.”*

Delete these sentences as you have stated the aims of the article above and these sentences are repetitive.

**In “Historical context of opal mining in Ethiopia”**

 What is mentioned in this thread is an expansion of what is mentioned in the “Introduction”. To avoid repetition, those in the “Introduction” can be deleted. Or this sub-heading can be combined with similar sentences in the “Introduction”.

**In “3.8.Ethics statement”**

 Almost the same text as in this heading appears at the end of the article, i.e. above the “References”. This heading should therefore be deleted here. However, if there is a different sentence, you can add it below. You can write “Annex 1” in front of the heading “List of declarations: Ethics and consent”.

**In “4.1. Challenges of traditional opal mining in North wollo”**

 •  You can replace *“The first category comprises challenges”* with *“The first category comprises* *technical challenges”.*

 • You mentioned that occupational accidents are a detrimental factor to opal mining in Ethiopia. Occupational accidents increase mining costs and cause loss of profit for mining companies. (If it would disrupt the flow of this thread, it would be useful to address this issue in a “Discussion” section as factors that impair the feasibility of mining)

 • You have stated in different parts of the article that opal production cannot be made technically and economically. In the world, the feasibility of ornamental stones can be determined professionally. You can refer to the examples in Literature.

 • In Ethiopia, where small-scale mining is practiced, you mentioned the inefficiency of production in many fields. At this point, could basin mining instead of small-scale field mining contribute to making opal mining in Ethiopia more feasible? This possible higher contribution is based on the production of opal reserves identified by more technical reports of the cooperatives in Ethiopia you mentioned. You can review in Literature on this subject.

 • *“15,000* *to* *40,000* *ETB per kilogram”*

 The article refers to both the US dollar and the “ETB”. How many ETB is one US dollar? Can you share this information by referencing it in a footnote at the appropriate place in the article where ETB is mentioned?

 • I suggest that you discuss the problems of mine management in a sub-heading or in an appropriate place close to the "Discussion" section of your article, taking into account the practices of other countries. Of course, the practices of each country are different from each other. However, the suggestions given for solving the problems in some countries may also be useful for solving the problems in Ethiopia. Because, in order to increase the incomes of households in mining areas, more feasible mining operations will be needed. Opening more or higher capacity mining operations could also increase household incomes. In this regard, you can also use the problems of mining companies in Turkey, for example, in the "Discussion" in the article, although some of them are not similar to the practices in Ethiopia. Turkey is an example of mining practices in other developing countries because of the problems faced by mining and the proposed solutions to these problems. Mining operations in Turkey pay many land use fees. This weakens the feasibility of mining operations. In addition, the high number of institutions and bureaucracy in obtaining mining permits can reduce the attractiveness of investments and result in the abandonment of active mining licenses. This situation directly affects the incomes of the people in the mining region.

 • You can benefit from the studies in Literature on the negative impact of mining permits and bureaucracy on mining investments.

 • You can benefit from the studies in literature on land use costs and incentivizing mining.

 • Do land use fees in Ethiopia negatively affect the feasibility of mining? What are the fees and types of fees charged by mining companies? Do you think these fees negatively affect households in the mining area? Or is it necessary to reduce these fees in order to increase household incomes? Or which of these royalties, and to what extent, could be beneficial to pass on to local people? You can use the studies in the literature on the share of mining royalties given to local communities in the “Discussion” of the paper.

 • For example, in Turkey, mining licenses can be put as capital in commercial companies. This is a factor that can increase the power of companies. Of course, there are other factors that can increase the capital power of companies. You can also develop a proposal on whether all these factors can increase the power of mine operators in Ethiopia. In particular, is there a minimum capital required by legislation for mining in Ethiopia? This minimum capital varies according to mineral groups in Turkey.

 • You have analyzed the impact of mining on household incomes in your article. At this point, the question arises as to how many workers are employed in each mine and how much they are paid. You have shared some numbers on this issue. On the other hand, it would be useful to make use of the studies in Literature to increase the salaries of workers or engineers working in mines.

**In “Recommendations”**

 The heading "Recommendations" is very valuable for solving the problems of opal mining in Ethiopia that you mentioned. However, these recommendations need to be filled in. In other words, is the technical, social, and economic capacity sufficient for each recommendation to be implemented in Ethiopia? In this context, a "Discussion" needs to be made by giving examples from other countries' practices in the literature. The "Discussion" should precede the "Conclusions" and "Recommendations". Subheadings can be made for the "Discussion" using what I have suggested above. These headings can be created not only from the existing "Recommendations" but also by adding factors that affect opal mining such as mining permits, land use fees, royalties, taxes, etc. that negatively affect investment attractiveness. A brief mention of these would not be a departure from the subject of the article. Making opal mining more feasible and attractive for investors will have a direct or indirect positive impact on the incomes of the people of the region.

 The authors of the article can use all the sentences above as their own sentences (while developing their articles) in their articles.

 I wish you success.

Is the work clearly and accurately presented and does it cite the current literature?

Partly

If applicable, is the statistical analysis and its interpretation appropriate?

Yes

Are all the source data underlying the results available to ensure full reproducibility?

Yes

Is the study design appropriate and is the work technically sound?

Yes

Are the conclusions drawn adequately supported by the results?

Yes

Are sufficient details of methods and analysis provided to allow replication by others?

Yes

Reviewer Expertise:

AREA OF INTERESTS•          Mining legislation•          Mining exploration and operation projects•          Mineral economics, resources economics•          Costs and cost analysis, cost benefit analysis•          Natural resources rents, taxation•          Mining and energy/infrastructure investments conflicts•          Land acquisition and land use in mining, land use change, compensation, evaluation of abandoned mining areas in terms of environment and urbanism, industrial heritage•          Mining waste management, rehabilitation cost•          Occupational health and safety in mines, supervising of mines.

I confirm that I have read this submission and believe that I have an appropriate level of expertise to confirm that it is of an acceptable scientific standard, however I have significant reservations, as outlined above.

# Response

**Published:** 2025-01-27

**Authors:** Tadesse Wudu Abate

**Affiliations:** Economics, Woldia University, Weldiya, Amhara, Ethiopia

Dear Taşkın Deniz Yıldız, Thank you for the valuable and expertise comments you provided for this articles.  we have revised the article based on you comments but failed to consider some variable yue suggested to be included because we didn't collected data on those issues. we will conduct a research in near future by adding factors that affect opal mining such as mining permits, land use fees, royalties, taxes, etc. Thank You Sir.
